# The Role of Estrogen Receptor β (ERβ) in the Establishment of Hierarchical Social Relationships in Male Mice

**DOI:** 10.3389/fnbeh.2018.00245

**Published:** 2018-10-22

**Authors:** Mariko Nakata, Anders Ågmo, Shoko Sagoshi, Sonoko Ogawa

**Affiliations:** ^1^Laboratory of Behavioral Neuroendocrinology, University of Tsukuba, Tsukuba, Japan; ^2^Research Fellow, Japan Society for Promotion of Science (JSPS), Tokyo, Japan; ^3^Department of Psychology, University of Tromsø, Tromsø, Norway

**Keywords:** gonadal steroid hormone, testosterone, aggressive behavior, dominance hierarchy, rank determination, agonistic behavior, social interaction, tube test

## Abstract

Acquisition of social dominance is important for social species including mice, for preferential access to foods and mates. Male mice establish social rank through agonistic behaviors, which are regulated by gonadal steroid hormone, testosterone, as its original form and aromatized form. It is well known that estrogen receptors (ERs), particularly ER α (ERα), mediate effects of aromatized testosterone, i.e., 17β-estradiol, but precise role played by ER β (ERβ) is still unclear. In the present study, we investigated effects of ERβ gene disruption on social rank establishment in male mice. Adult male ERβ knockout (βERKO) mice and their wild type (WT) littermates were paired based on genotype- and weight-matched manner and tested against each other repeatedly during 7 days experimental period. They underwent 4 trials of social interaction test in neutral cage (homogeneous set test) every other day. Along repeated trials, WT but not βERKO pairs showed a gradual increase of agonistic behaviors including aggression and tail rattling, and a gradual decrease of latency to social rank determination in tube test conducted after each trial of the social interaction test. Analysis of behavioral transition further suggested that WT winners in the tube test showed one-sided aggression during social interaction test suggesting WT pairs went through a process of social rank establishment. On the other hand, a dominant-subordinate relationship in βERKO pairs was not as apparent as that in WT pairs. Moreover, βERKO mice showed lower levels of aggressive behavior than WT mice in social interaction tests. These findings collectively suggest that ERβ may play a significant role in the establishment and maintenance of hierarchical social relationships among male mice.

## Introduction

Individuals of social species often establish hierarchical social relationships with their conspecifics. Once their social rank is determined, dominant (higher rank) individuals can get preferential access to resources including food, territory and mates. It is known that group-housed male mice establish a social hierarchy both in the wild and in laboratory housing conditions. Their social order is determined through agonistic interactions, which include not only active aggressive behavior but also various behavioral responses to opponents’ aggression, such as fleeing, immobility and upright submissive posture (Grant and Mackintosh, 1963). Once hierarchy is settled, a highest rank male mouse (α-dominant male) consistently attacks subdominant, subordinate and intruder males and successfully defends his territory from rivals (Singleton and Hay, 1983; Palanza et al., 1996; Miczek et al., 2001; Wang et al., 2011).

For assessment of social rank among mice, various testing paradigms, such as direct observation of agonistic behaviors (Miczek et al., 2001) and tube test (Wang et al., 2011), have been developed. Previous studies on neural mechanisms underlying the establishment of social hierarchy in male mice revealed that the gonadal steroid hormone, testosterone, plays an essential role (Machida et al., 1981; Giammanco et al., 2005). Testosterone is mainly secreted from the testes into the blood stream and binds not only to androgen receptors but also to estrogen receptors (ERs), after conversion to 17β-estradiol by aromatase in the brain. Two subtypes of nuclear ERs, ERα and ERβ, are known to mediate intracellular actions by 17β-estradiol, aromatized testosterone.

It is well established that ERα is necessary for the induction of male sexual and aggressive behaviors in mice (Ogawa et al., 1997, 1998, 2000; Rissman et al., 1997; Wersinger et al., 1997). In contrast, the role of ERβ in the regulation of male social behaviors is still not fully understood. ERβ has been thought to modulate male social behaviors in a complex manner, rather than simply induce a stereotyped behavioral pattern. Ogawa et al. (1999) initially reported altered aggressive behavior in male ERβ knockout (βERKO) mice. In aggression tests using a resident-intruder paradigm, wild-type (WT) mice showed a gradual increase of aggression levels over three consecutive tests. On the other hand, βERKO males showed high levels of aggression (longer duration of aggressive bouts) starting on the first trial and kept steady levels of aggression throughout the repeated tests. Moreover, βERKO males were much more aggressive particularly during pubertal period compared to WT mice (Nomura et al., 2002; Handa et al., 2012; Tsuda et al., 2014). These experience- and age-dependent influences of ERβ gene disruption suggested that ERβ might regulate male social behaviors in a specific context such as establishment of social hierarchy.

In the present study, we investigated the influence of ERβ gene disruption on the process of establishment of a hierarchical social relationship among socially naïve mice. βERKO and WT male mice were paired with same-sex and same-genotype individuals. Agonistic and prosocial behaviors were analyzed in social interaction tests performed repeatedly (4 trials) over 7 days. Social rank was also assessed with the use of the tube test, right after each trial of the social interaction test. In order to identify critical behavioral acts between paired mice for the determination of social rank, we also analyzed behavioral transition patterns and compared these between WT pairs and βERKO pairs.

## Materials and Methods

### Subjects

Gonadally intact and sexually naïve male βERKO and WT littermate mice (βERKO: 12 pairs, *n* = 24, WT: 10 pairs, *n* = 20) were used as experimental animals. They were obtained from a breeding colony maintained at the University of Tsukuba. Original breeding pairs were provided by Dr. KS Korach at the National Institute of Environmental Health Sciences (Research Triangle Park, NC, USA) and completely backcrossed to C57BL/6J mice (Krege et al., 1998). Mice were weaned at 3 weeks of age and then group housed with same-sex littermates in genotype-mixed manner. They were kept in polypropylene clear plastic cages (19 × 29 × 12 cm) until the experiment started. They were kept under standard housing conditions (23 ± 2°C, 12:12 light/dark cycle with lights off at 12:00). Food and water were provided *ad libitum*. All procedures were conducted in accordance with the National Institutes of Health guidelines and were approved by the Animal Care and Use Committee and the Recombinant DNA Use Committee at the University of Tsukuba. All efforts were made to minimize the number of animals and their suffering.

### Experimental Procedures

Starting at 17 ± 4.5 weeks old and throughout the experiment, all mice were individually housed in small transparent plastic home-cages (12.5 × 20 × 11 cm). Non-littermate mice from the same genotype and matched body weight (±3.5 g) were paired (homogeneous pair) and tested against each other throughout the experiment. After 1 week of individual housing, mice were trained for the tube test on two consecutive days. Starting on the next day, each pair underwent the social interaction test followed by the tube test on every other day (days 1, 3, 5 and 7) for a total of 4 trials (trials 1, 2, 3 and 4). All behavioral tests were recorded using digital video cameras and scored by an experimenter unaware of the animals’ experimental group, using a digital event recorder program (Recordia 1.0b, O’Hara & Co., Ltd.).

### Social Interaction Test

Social interaction behaviors between the paired mice were assessed in a neutral testing cage (19 × 29 × 12 cm) for 15 min. All tests were done under red light illumination during the dark phase of the light/dark cycle. At first, the testing cage was divided into two compartments by inserting a black Plexiglas board (divider) at the middle of the cage and mice were habituated in each compartment for 5 min. At the beginning of the test, the divider was removed and social interaction behaviors were observed. The cumulative number and duration of aggression, fleeing, immobility, upright submissive posture, approach, sniffing, huddling and grooming were recorded. Aggression was defined as a series of behavioral interactions consisting of at least one of the following: chasing, boxing, wrestling, biting and offensive lateral attack, often accompanied by biting. The cumulative number of tail rattling was also recorded. These nine behavioral acts were classified into two groups for further analysis: agonistic behaviors (aggression, fleeing, immobility, submissive posture and tail rattling) and prosocial behaviors (sniffing, grooming, approaching and huddling). Sniffing and grooming were further categorized into face-targeted or body-targeted. Thereafter, ratio of the face-targeted was calculated and compared between the ranks determined by the tube test. Sniffing and grooming were combined for this analysis (see Supplementary Table S1).

### Tube Test

Right after the completion of the social interaction test, the tube test was conducted to assess social rank between the paired mice. All tests were done under red light illumination during the dark phase of the light/dark cycle. A clear plexiglass tube (3 cm inner diameter and 45 cm long) was placed at the center of the testing arena (70 × 50 cm) surrounded by black wall (20 cm). Starting from 2 days before the first social interaction test, all mice were trained individually to run through the tube from one end to the other eight times per day for two consecutive days, as previously described (Wang et al., 2011). A black plastic escape box (13 × 14 × 13 cm) was placed at the end of the tube during these training sessions.

On each testing day, all mice were individually given two pre-test trials to run through the tube without an escape box. In test trials, mice in each pair were released simultaneously from one of two ends of the tube. Each test trial lasted until one mouse forced the other to retreat from the tube. The former mouse remaining in the tube was judged as a “winner” and the latter mouse ejected from the tube was judged as a “loser”. The winner animal ID and latency to loser ejection were recorded in each test trial. Winner shift, defined as the winner being different between two consecutive test trials (Oakeshott, 1974; Wang et al., 2011), was also analyzed. Since video recordings of three pairs (two WT and one βERKO pairs) on Day 1 failed, all data from these pairs were excluded from the analysis.

### Analysis of Behavioral Transition During Social Interaction Tests

Behavioral transitions of two consecutive behavioral events occurring with an interval of less than 6 s were analyzed. They were classified as monad or dyad transitions depending on actor(s) of the behavioral events. Among nine behavioral acts, upright submissive posture was excluded from this analysis because only limited mice showed this behavior. In monad transitions, actors of the two consecutive behavioral events were the same mouse. In dyad transitions, an actor of the first behavioral event (initiator) and that of the second behavioral event (responder) were different mice. For monad transitions, the probabilities of transitions were calculated and 8 kinetograms were constructed for each trial and genotype. For analysis of dyad transitions, eight behavioral events recorded in social interaction test was partially combined as follows; subordinate behaviors including fleeing and immobility, and prosocial behaviors including sniffing, grooming and huddling. Probabilities of dyad transitions were then calculated and 8 kinetograms were constructed for each test, genotype and rank (winner or loser in the tube test). Differences between tests, genotypes and ranks were analyzed qualitatively based on the diagrams. For dyad transitions, the number of all transitions, transitions initiated with approach, and transitions responded with subordinate behaviors were also counted and statistically analyzed.

### Statistics

Agonistic and prosocial behaviors in social interaction tests, and latency to loser ejection in tube tests were analyzed by a two-way analysis of variance (ANOVA), repeated measurements of the main effects for genotype, trials and their interaction. *Post hoc* power analyses for the main effects and their interaction of ANOVAs (Cohen, 1992) were conducted with G*Power version 3.1.9.2 (Faul et al., 2007). Genotype differences in percentage of animals showing aggression in social interaction tests, winner shift frequency of tube tests, and winner/loser ratio of initiator of dyad transitions were analyzed by a Fischer’s exact test, with stratified analysis of Benjamini and Hochberg method. Genotype differences in total number of all dyad transitions were analyzed by a Chi-squared test with stratified analysis of Benjamini and Hochberg method. Rank differences in approach- or subordinate-transitions were analyzed by a Binomial test. ANOVAs were conducted using the SPSS version 21 (SPSS Inc., Chicago, IL, USA). Fischer’s exact test, Chi-squared test and Binomial test were conducted with js-STAR (v. 8.0.0 j) software. Statistically significant differences were considered at *p* < 0.05.

## Results

### Agonistic and Prosocial Behaviors in the Social Interaction Test

**Figure 1:**
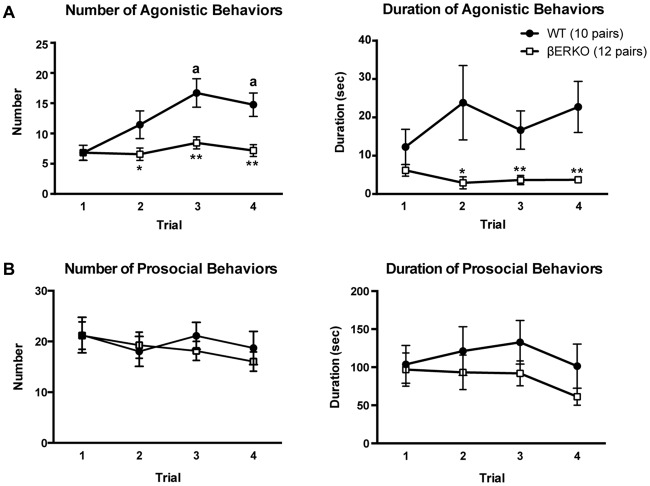
Influence of estrogen receptor β (ERβ) gene disruption on agonistic and prosocial behaviors in the homogeneous set social interaction test. **(A)** Unlike wild type (WT; ●), ERβ knockout (βERKO; □) mice did not show an increase in the number (left panel) of agonistic behaviors over trials. Moreover, βERKO mice showed shorter duration of agonistic behaviors (right panel). **(B)** There was no difference between βERKO and WT groups in the number (left panel) and duration (right panel) of prosocial behaviors. All data are presented as mean ± SEM. ^a^*p* < 0.01 compared with trial 1 of the same genotype; *p < 0.05; **p < 0.01 compared with WT in the same trial.

The cumulative number of agonistic behaviors gradually increased over the four trials in WT mice, but did not change in βERKO mice (Figure 1A, left panel; genotype: *F*_(1,42)_ = 14.741, *p* < 0.001, *d* = 0.592, power (1-β) = 0.997; trial: *F*_(3,126)_ = 6.333, *p* < 0.001, *d* = 0.388, power (1-β) = 0.999; genotype × trial: *F*_(3,126)_ = 3.617, *p* = 0.015, d = 0.293, power (1-β) = 0.998). In WT mice, the number of agonistic behaviors in trials 3 and 4 were significantly higher than in trial 1 (*p* < 0.01), whereas no significant difference was observed between trials in βERKO mice. Moreover, βERKO mice showed a significantly lower number of agonistic behaviors, compared to WT pairs in trial 2 (*p* < 0.05), and trials 3 and 4 (*p* < 0.01), although there was no genotype difference in trial 1. βERKO mice also showed significantly a shorter overall cumulative duration of agonistic behaviors, compared to WT mice (Figure 1A, right panel; genotype: *F*_(1,42)_ = 8.629, *p* = 0.005, *d* = 0.453, power (1-β) = 0.903). Significant genotype difference was observed in trial 2 (*p* < 0.05), and trials 3 and 4 (*p* < 0.01), although there was no significant main effect of trial and interaction of genotype and trial (trial and genotype × trial, n.s.). In contrast, both number and duration of prosocial behaviors were not different between genotypes and did not change over the four trials (Figure 1B; main effects of genotype, trial, and interaction, n.s.). Additionally, detailed analysis of sniffing and grooming revealed that in both WT and βERKO pairs, there was no difference in the probability of face-targeted sniffing and grooming between the winner and loser in the tube test conducted in the same experimental day following the social interaction test (Supplementary Table S1).

### Tube Test

**Figure 2:**
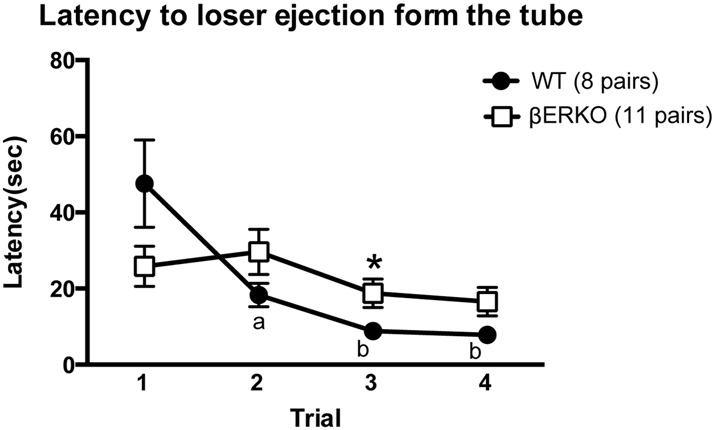
Influence of ERβ gene disruption on the latency to loser ejection in the tube test. Unlike WT (●), βERKO (□) mice did not show a decrease in the latency to loser ejection over trials. Data are presented as mean ± SEM. ^a^*p* < 0.05; ^b^*p* < 0.01 compared with trial 1 of the same genotype; *p < 0.05 compared with WT in the same trial.

The latency to loser ejection in tube tests decreased significantly during the four test trials in WT but not in βERKO pairs (Figure 2; trial: *F*_(3,51)_ = 9.143, *p* < 0.001, *d* = 0.733, power (1-β) = 0.999; genotype × day: *F*_(3,51)_ = 8.551, *p* = 0.007, *d* = 0.709, power (1-β) = 1.000; genotype: n.s.). As for the winner shift, there was a trend of a gradual decrease from trial 2 to trial 4 only in WT, but not in βERKO mice, although there were no statistically significant genotype differences (Table 1; trials 2, 3 and 4; n.s.).

**Table 1:** Number of pairs with winner shift in each trial.

Trial		2	3	4
WT	Shift	5	3	2
	No Shift	5	7	8
βERKO	Shift	3	4	2
	No Shift	9	8	10

*There was no significant genotype difference in frequency of the winner shift in each trial*.

### Monad Behavioral Transition Patterns During Social Interaction Tests

**Figure 3:**
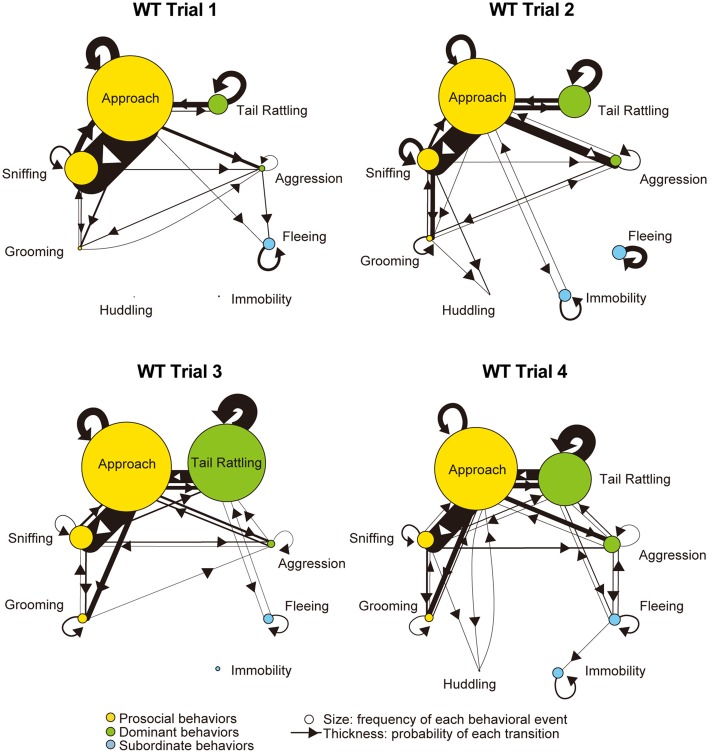
Kinetograms for monad behavioral transitions of WT mice in each trial. The diameters of circles were calculated for each genotype and trial using the following formula. Circle diameter is proportional to (Total number of each behavioral event)/(Total number of all behavioral events within each genotype and trial). Type of behavioral events (prosocial, dominant or subordinate) were indicated by circle color. Width of the arrows were also calculated using the following formula. Arrow width is proportional to (Total number of each transition)/(Total number of all transitions within each genotype and trial). Arrowhead indicates direction of each transition.

**Figure 4:**
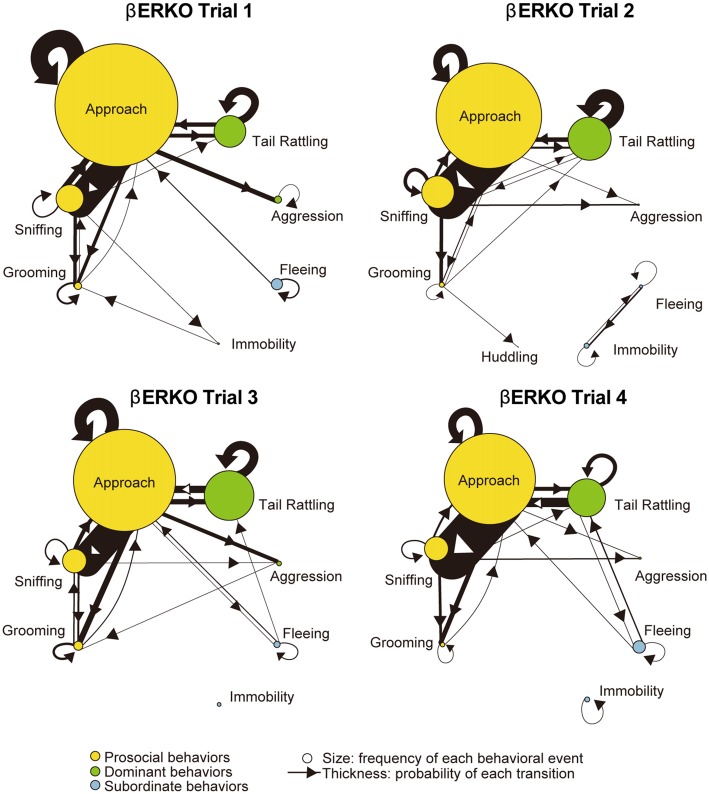
Kinetograms for monad behavioral transitions βERKO mice in each trial.The diameters of circles were calculated for each genotype and trial using the following formula. Circle diameter is proportional to (Total number of each behavioral event)/(Total number of all behavioral events within each genotype and trial). Type of behavioral events (prosocial, dominant or subordinate) were indicated by circle color. Width of the arrows were also calculated using the following formula. Arrow width is proportional to (Total number of each transition)/(Total number of all transitions within each genotype and trial). Arrowhead indicates direction of each transition.

Monad-type behavioral transition patterns during social interaction tests, in which two consecutive behavioral events were acted by the same mouse, were visualized using kinetograms for each genotype and trial. To construct each kinetogram, data from all mice were combined. Kinetograms for WT mice (Figure 3) indicated that WT mice mainly showed investigative behavior, particularly transitions from approach to sniffing, in trial 1. Along repeated trials, behavioral transition patterns of WT mice shifted from investigation to threatening which includes tail rattling. Significant increases of aggression and tail rattling in trials 3 and/or 4 (Supplementary Figure S1A) were consistent with these behavioral changes in WT mice. In contrast, βERKO mice did not show any obvious changes of their behavioral patterns throughout the four trials and mainly exhibited investigative behavior (Figure 4).

### Analysis of Dyad Social Interaction Patterns Between Winners and Losers

To examine social interaction between winners and losers, dyad behavioral transitions, in which one mouse (responder) responded to a preceding behavioral event acted by the other mouse (initiator), were analyzed.

The total number of dyad transitions, as an index of richness of social interaction, was first examined and compared between WT and βERKO pairs (Table 2). WT pairs showed a gradual increase of the number of dyad transitions along repeated trials whereas βERKO pairs did not show such changes. Statistical analysis revealed that the total number of all dyad transitions of βERKO pairs was not different from that of WT pairs on trial 1, but significantly fewer in trials 3 and 4 (Table 2; trial 2: ${\text{X}}_{\left(1\right)}^{2}$ = 2.976, 0.050 < *p* < 0.100; trial 3: ${\text{X}}_{\left(1\right)}^{2}$ = 25.638, *p* < 0.010; trial 4: ${\text{X}}_{\left(1\right)}^{2}$ = 23.554, *p* < 0.010). It should be noted that throughout the 4 trials, the numbers of dyad transitions initiated by winners and losers were roughly equal in both genotypes (Table 2; trials 1, 2, 3 and 4; n.s.).

**Table 2:** Total numbers of dyad transitions in each trial and genotype group.

	Initiator	1	2	3	4
WT	Winner	55	73	102	99
	Loser	52	63	105	101
	Total	107	136	207	200
βERKO	Winner	71	54	65	62
	Loser	55	55	51	52
	Total	126	109	116**	114**

Estrogen receptorβ knockout (βERKO) mice showed a significantly lower number of dyad transitions in trials 3 and 4 than wild type (WT) mice, when winners and losers were combined. Data are presented as total of each trial and group. **p < 0.01 compared with WT in the same trial.

**Figure 5:**
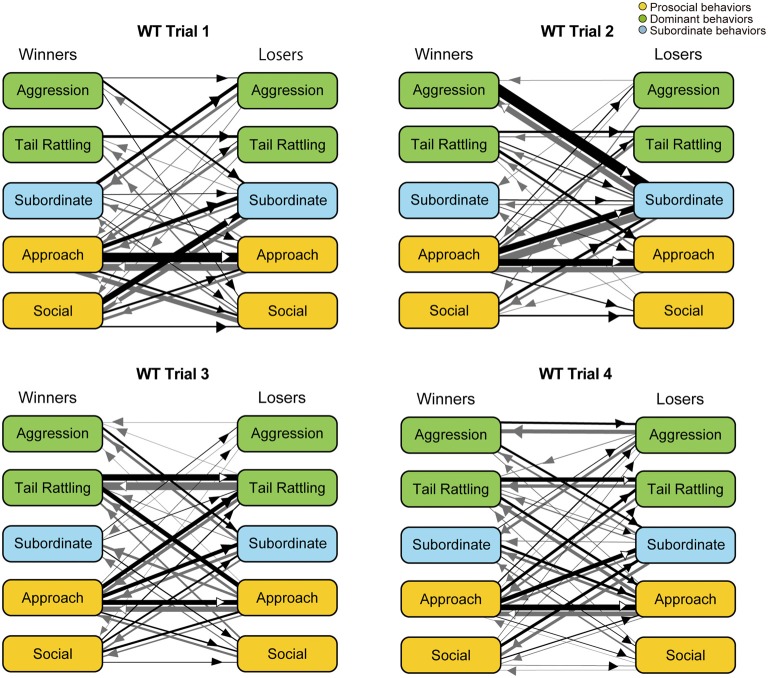
Kinetograms for dyad behavioral transitions of WT pairs in each trial. Width of the arrows were also calculated using the following formula. Arrow width is proportional to (Total number of each transition)/(Total number of all transition within each genotype, trial and initiator). Black arrows indicate dyad transitions initiated by the winners. Gray arrows indicate dyad transitions initiated by the losers.

Further analysis using kinetograms revealed that in WT pairs approach responded by approach (approach—approach) was a predominant type of dyad transitions in trial 1 (Figure 5). However, WT winners showed one-sided aggression thereafter. In trial 2, a strong asymmetry pattern of dyad transitions, initiated by winners’ aggression or approach and followed by losers’ subordinate behaviors, became obvious. Consistent with the findings in monad transition analysis, transitions as tail rattling—tail rattling and approach—tail rattling became predominant in trials 3 and 4.

**Figure 6:**
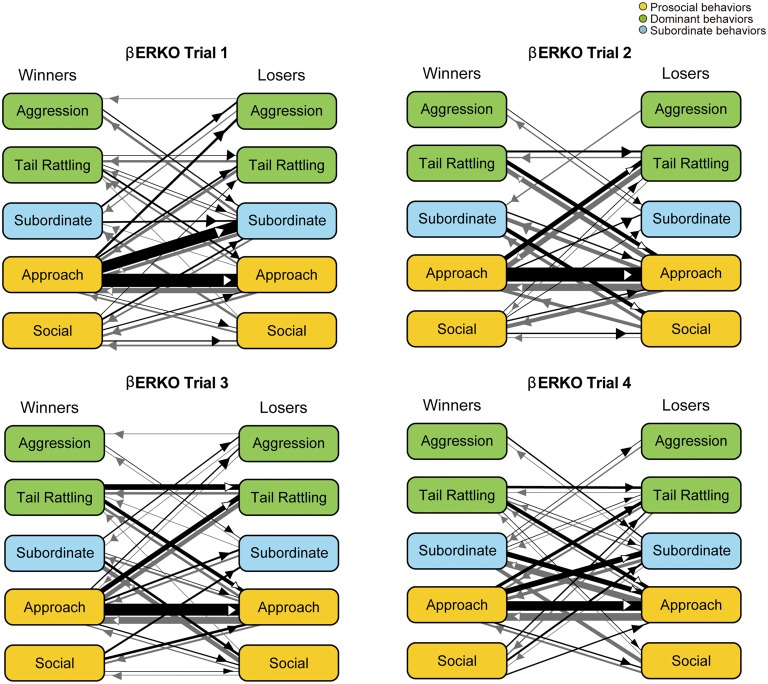
Kinetograms for dyad behavioral transitions of βERKO pairs in each trial. Width of the arrows were also calculated using the following formula. Arrow width is proportional to (Total number of each transition)/(Total number of all transition within each genotype, trial and initiator). Black arrows indicate dyad transitions initiated by the winners. Gray arrows indicate dyad transitions initiated by the losers.

In contrast to WT pairs, the most predominant transition was approach—approach in βERKO pairs in all four trials (Figure 6). In trial 1, an asymmetry transition pattern in which winners’ approach was followed by losers’ subordinate behavior, was observed. Starting with trial 2 and thereafter, most of the transitions were symmetrical between winners and losers.

The number of transitions initiated by approach (approach-transitions; Table 3) and those responded by subordinate behaviors (subordinate-transitions; Table 4) were counted separately for winners and losers in each trial. Statistical analysis revealed that WT winners initiated more approach-transitions than WT losers in trial 2 whereas βERKO winners did so in trial 1 (Table 3; WT, trial 2; *p* = 0.016; βERKO, trial 1; *p* < 0.001, Binomial test). Consistent with these findings, losers of WT pairs showed a significantly higher number of subordinate behavior than winners in trial 2 (Table 4; WT, trial 1; *p* = 0.087, trial 2; *p* < 0.001, trial 3; *p* = 0.080 in Binomial tests). On the other hand, losers of βERKO pairs showed a significantly higher number of subordinate behaviors than winners in trial 1 (Table 4; βERKO, trial 1; *p* = 0.002, Binomial tests).

**Table 3:** Total numbers of dyad transitions initiated with approach (approach-transition).

	Initiator	1	2	3	4
WT	Winner	24	31*	42	41
	Loser	19	14	34	33
βERKO	Winner	50**	30	34	31
	Loser	17	30	21	24

In WT pairs, winners initiated significantly more approach-transition than losers in trial 2. On the other hand, this rank asymmetry in βERKO pairs was observed in trial 1. Data are presented for each trial, genotype and rank. *p < 0.05; **p < 0.01 compared with losers in the same genotype.

**Table 4:** Total number of dyad transitions ended by subordinate behaviors (subordinate-transition).

	Responder	1	2	3	4
WT	Winner	9	4	11	20
	Loser	19	40**	22	27
βERKO	Winner	7	13	11	19
	Loser	25**	4*	7	15

In WT pairs, losers responded with subordinate behaviors more frequently than winners in trial 2. On the other hand, losers in βERKO pairs did so in trial 1. Data are presented for each trial, genotype and rank. *p < 0.05; **p < 0.01 compared with winners in the same genotype.

## Discussion

In the present study, we investigated the effects of ERβ gene disruption on the establishment of hierarchical social relationships among male mice. We assessed behavioral changes during repeated trials in social interaction tests conducted in neutral cages (homogeneous set test) followed by tube tests for social rank evaluation.

Over four trials, WT pairs showed a gradual increase in agonistic behaviors, such as aggression and tail rattling in social interaction tests. In tube tests, a corresponding decrease of latency to loser ejection was observed. After an initial investigative period in trial 1, WT winners defeated the losers and acquired their dominance. Detailed analysis of behavioral transitions revealed that an asymmetry in behaviors of the winners and losers appeared in trial 2 in WT pairs—i.e., losers responded to winners’ one-sided attack with subordinate behaviors. Summarized kinetograms (Supplementary Figure S2) clearly demonstrate one-sided dyad transitions from winners’ aggression and/or tail rattling to losers’ subordinate behaviors in trial 1 and 2 (indicated by thickness of black lines in the left top kinetogram). In trials 3 and 4, winners tried to defend their dominance status through continuous agonistic interactions including frequent tail rattling (indicated with large size circles and thick transition lines in the left bottom kinetogram of Supplementary Figure S2). Occurrence of aggressive behavior and winner shifts, even in trial 4, suggested that not all WT pairs successfully established stable dominant-subordinate relationships by the end of four trials. Observation of upright submissive posture also supports these hypotheses (Supplementary Figure S3). In WT group, four mice in three pairs showed submissive posture during the social interaction tests and three out of these four mice were losers in the tube test. As one exception, the winner of the pair W15 showed upright submissive posture in trial 4. However, in this trial, both winner and loser showed aggression as well. Additionally, in WT pairs, the results of the tube test reflect dominant-subordinate relationships in the social interaction test after trial 2, consistent with previously reported findings (Wang et al., 2011). These results collectively suggest that WT pairs went through a process of social rank establishment.

In contrast to the findings in WT pairs, βERKO pairs showed little behavioral changes throughout the four trials. Although rank asymmetry in behavioral patterns was observed in trial 1, apparent one-sided aggressive behavior was not observed in βERKO pairs. They kept investigating each other intensively without any increase of aggression or tail rattling throughout all four trials (Supplementary Figures S1, S2, right kinetograms). The total number of dyad transitions was significantly lower in βERKO pairs, compared to WT pairs in trials 3 and 4. Thus, a dominant-subordinate relationship in βERKO pairs was not as apparent as observed in WT pairs. Although some of βERKO mice showed submissive posture, it was not necessarily observed in losers in the tube test (Supplementary Figure S3). These results suggest that disruption of ERβ gene suppressed the expression of typical behavioral interactions necessary for progression of hierarchical social relationship establishment among male mice.

It has been reported previously that βERKO mice show a tendency of prolonged investigation of social stimuli (Handa et al., 2012; Tsuda et al., 2014). Consistent with these findings, βERKO pairs in the present study showed higher levels of social investigatory behavior, compared to WT mice throughout the four trials. These behavioral characteristics may be partly due to changes in the level of anxiety in βERKO mice. Several lines of evidence have suggested that ERβ plays a role in the estrogenic regulation of anxiety-related behaviors (Walf and Frye, 2007; Weiser et al., 2008; Tomihara et al., 2009). In male mice, treatment with specific agonist to ERβ decreased anxiety-related behavior in a non-social context (Frye et al., 2008).

Increased anxiety levels may also underlie the reduced aggressive behavior in βERKO mice during social interaction tests in the present study. βERKO mice showed a lower level of aggression and tail rattling than WT mice, although they were not different from WT in terms of other components of agonistic behaviors and prosocial behaviors (Supplementary Figure S1). Previous studies reported a consistent tendency of increased aggressive behavior in βERKO male mice in resident-intruder paradigm tests (Ogawa et al., 1999; Nomura et al., 2002; Handa et al., 2012; Tsuda et al., 2014). Additionally, although the levels of aggressive behavior were not affected, treatment with selective agonist of ERβ increased dominant behaviors of resident male mice (Clipperton Allen et al., 2010). It should be noted that in those experiments, test mice were presented with a stimulus mouse in their own territory. In addition, olfactory-bulbectomized or gonadectomized males, which rarely counterattack, were used as stimulus animals. Therefore, it is assumed that ERβ activation suppress aggressive behavior in male mice in their own territory toward a stimulus animal which is less likely to counterattack—i.e., in case of an α-dominant male mouse. On the other hand, the homogeneous set test used in the present study provides a completely different ecological situation from the resident-intruder paradigm test. Since both mice in the pairs had intact gonads and olfactory bulbs, they were more likely to counterattack compared to stimulus animals in the resident-intruder paradigm test. Moreover, the social interaction test was conducted in a neutral cage, which was the territory of neither mouse. Thus, when the experimental animal is not in an advantageous situation—i.e. not an α-dominant male in his own territory—ERβ activation may be necessary to induce aggressive behavior and tail rattling for the establishment of a dominant-subordinate relationship.

In the present study, βERKO pairs showed little enhancement of aggression through repeated encounters. This behavioral phenotype is consistent with the previous studies with the resident-intruder paradigm tests (Ogawa et al., 1999). Decreased responsivity to repeated aggressive encounter may alternatively explain disruption of rank establishment in βERKO pairs. However, lack of behavioral change throughout the trials and active behavioral interactions toward rank establishment in βERKO mice may not be due to disruption in social memory and/or social recognition induced by ERβ gene disruption. To establish a social relationship through repeated behavioral interaction, mice need to recognize their opponents and keep social memory until the next trial. It was reported that βERKO male mice possess long-term social memory and are able to discriminate two male stimulus mice in social recognition tests (Sánchez-Andrade and Kendrick, 2011). It should be noted, however, in the present study, mice were group-housed with same-sex littermates until the experiments started. Thus, we cannot exclude the possibility that experience such as social defeat by a cage-mate during group-housed period may be different between genotypes and strengthen the effects of ERβ gene disruption.

In summary, we found that ERβ gene disruption may prevent social rank establishment among male mice. Unlike previously reported findings with the resident-intruder paradigm tests, βERKO male mice showed reduced levels of aggressive behavior in a neutral testing situation. It is hypothesized that ERβ activation may promote aggressive behavior in male mice to acquire social dominance until they establish the status as an α-dominant male. After that, ERβ activation may inhibit excess aggressive behavior by an α-dominant male to avoid further unnecessary injuries of subordinates. In addition, these behavioral phenotypes of βERKO male mice are observed in dyad tests (interaction between two mice). In future studies, it is needed to investigate further whether the βERKO mice are able to establish hierarchical social relationships in larger groups since being in a pair and in a larger group induce different hormonal status in male mice (Williamson et al., 2017). Taken together, we believe that ERβ may be involved in facilitating both the establishment and maintenance of hierarchical social relationships among male mice by regulating aggressive behavior in a social status-depending manner. Further studies are necessary to determine possible underlying neural mechanisms of ERβ-mediated regulation of social behavior.

## Author Contributions

MN, AÅ, and SO designed research. MN, AÅ and SS performed research and analyzed data; MN and SO wrote the article.

## Conflict of Interest Statement

The authors declare that the research was conducted in the absence of any commercial or financial relationships that could be construed as a potential conflict of interest.
