# Supplementary material for: The Role of Estrogen Receptor β (ERβ) in the Establishment of Hierarchical Social Relationships in Male Mice
Source: Front Behav Neurosci. 2018 Oct 22;12:245. doi: 10.3389/fnbeh.2018.00245 (PMC6204783; doi:10.3389/fnbeh.2018.00245)
Supplement: Supplementary file 1 [file Table_1.pdf]

Supplementary Material

The Role of Estrogen Receptor β (ERβ) in the Establishment of Hierarchical Social Relationships in Male Mice.

Mariko Nakata, Anders Ågmo, Shoko Sagoshi, and Sonoko Ogawa*

*** Correspondence:** Sonoko Ogawa: ogawa@kansei.tsukuba.ac.jp

Supplemental Figure 1

A

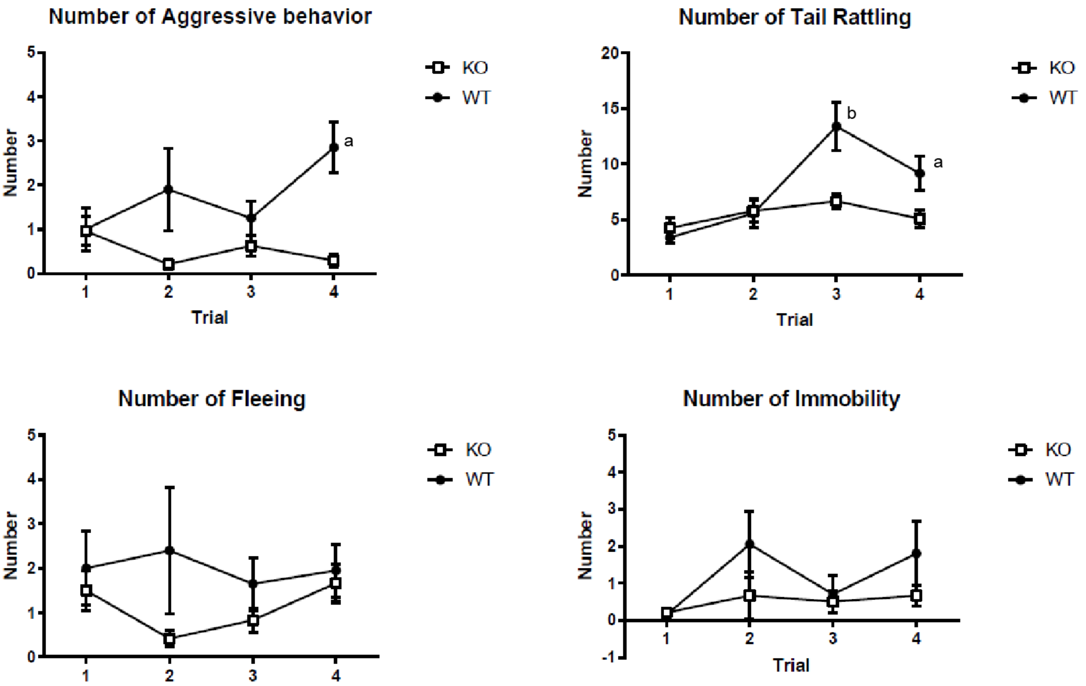

B

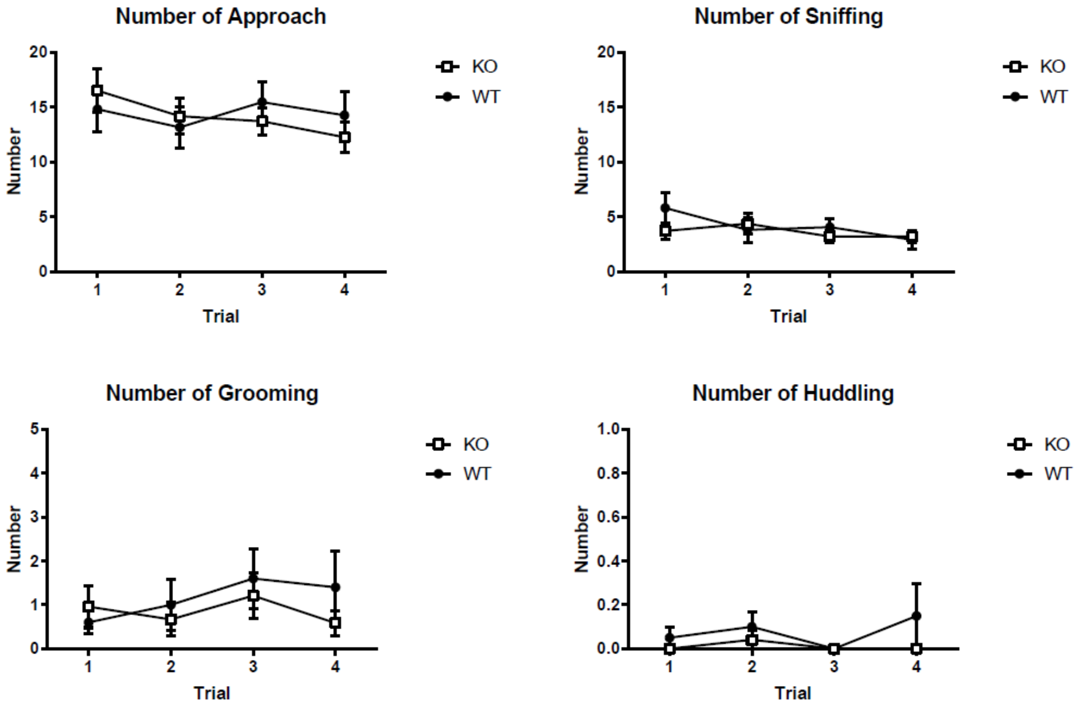

Figure S1. Influence of ERβ gene disruption on each behavioral events of agonistic behaviors in the homogeneous set social interaction test. (A) Cumulative number each behavioral events of agonistic behaviors. Unlike WT (●), βERKO (□) mice did not show increase of the number of aggressive behavior (top left panel; genotype: $F_{(1,42)} = 9.926$, $p = 0.003$; trial: $F_{(3, 126)} = 1.073$, n.s.; genotype x trial: $F_{(3, 126)} = 3.879$, $p < 0.011$, a: $p < 0.05$ vs trials 1 and 3) and tail rattling (top right panel; genotype: $F_{(1,42)} = 4.702$, $p = 0.036$; trial: $F_{(3, 126)} = 12.9$, $p < 0.001$; genotype x trial: $F_{(3, 126)} = 6.121$, $p = 0.006$) over trials. However, there was no difference between βERKO and WT groups in the number of fleeing (bottom left panel) and immobility (bottom right panel). (B) Cumulative number each behavioral event of prosocial behaviors. There was no difference between βERKO and WT groups in the number of prosocial behaviors including approach (top left panel), sniffing (top right panel), grooming (bottom left panel), and huddling (bottom right panel). All data are presented as mean ± SEM. Data were analyzed by a two-way analysis of variance (ANOVA) for repeated measurements for the main effects of genotype, trials and their interaction, and were conducted using GraphPad Prism 7 (GraphPad Software, Inc., San Diego, USA). a: $p < 0.05$ compared with trials 1 b: $p < 0.05$ compared with trials 1 and 2.

Supplemental Figure 2

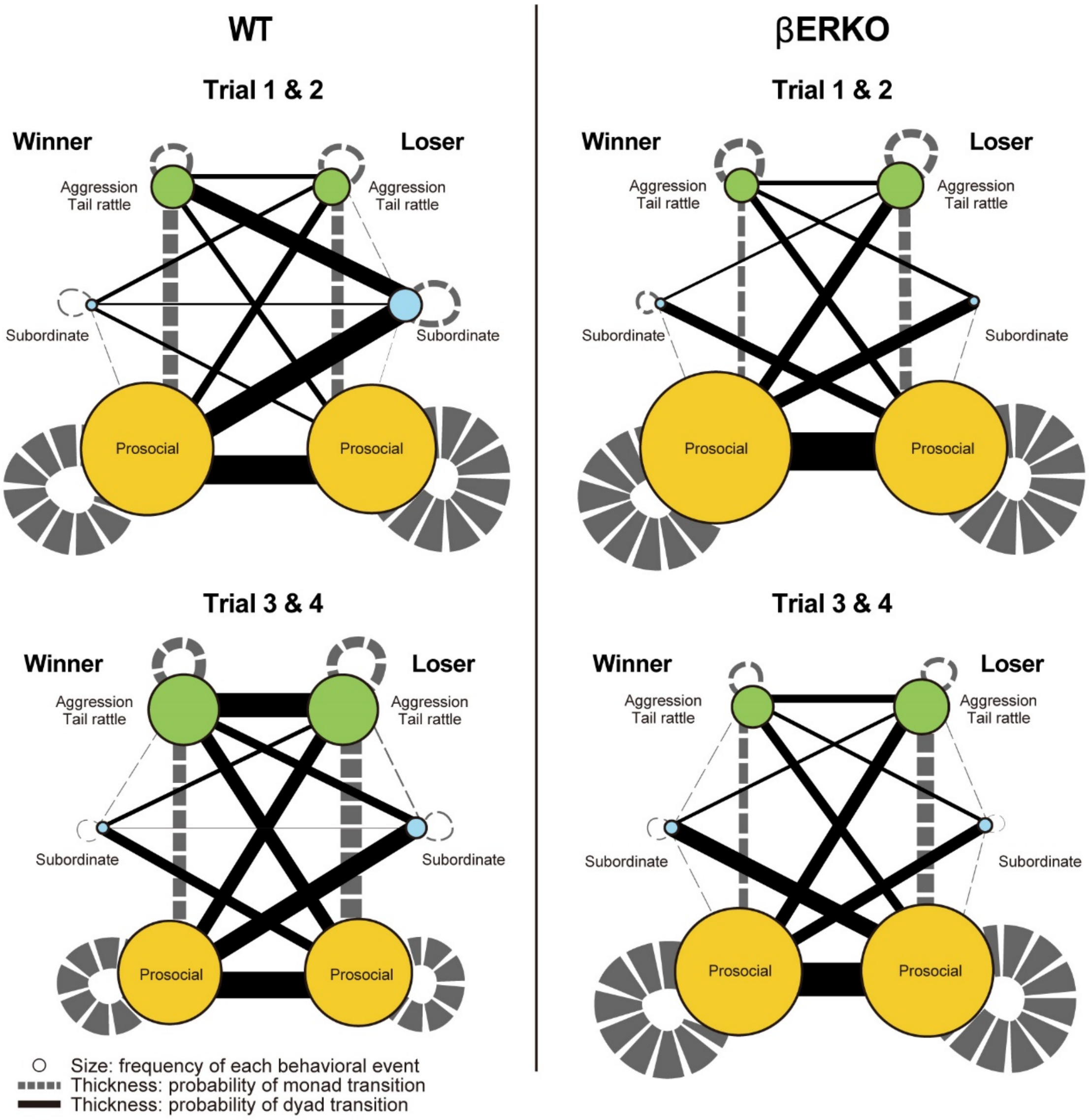

Figure S2 Summarized kinetograms for monad and dyad behavioral transitions.

To more clearly highlight genotype differences and changes of behavioral patterns, total number of the behavioral events in trials 1 and 2, and trials 3 and 4 were combined respectively. Winners and losers indicated in the figure were determined with the tube test conducted following the social interaction test. Circle diameter is proportional to (Total number of each category of behavioral events) / (Total number of all behavioral events within each genotype and trial). Aggression and tail rattling, and all prosocial behaviors including approach, sniffing, grooming and huddling, were combined respectively. Transitions were calculated as (Total number of each transition) / (Total number of all transitions within each genotype and trial) and resulting values were shown in lines with proportional width. Transitions between the two same behavioral categories with opposite directions were combined. Black lines indicate dyad transitions and gray dashed lines indicate monad transitions. In trial 1 and 2, WT pairs showed one-sided dyad transitions from winners' aggression and/or tail rattling to losers' subordinate behaviors (indicated by thick black lines). In trial 3 and 4, WT, but not βERKO, pairs showed an increase of aggression and/or tail rattling (indicated by circle size) and a relative increase of dyad transitions (indicated by thickness of transition lines).

Supplemental Figure 3

	WT						βERKO					
	W12		W15		W25		M15		M17		M21	
Trial	W	L	W	L	W	L	W	L	W	L	W	L
1	8						2			1		
2		16				16					4	
3		5		3			8					
4		12	2	2			3					

Figure S3. The upright submissive posture in the social interaction tests. The first row indicates genotypes and second row indicates pair IDs. W or L in the third row indicates winner (W) or loser (L) of the tube test conducted following the social interaction test. Number in each cell indicates number of submissive posture. In case of submissive posture was observed in more than one trial in each pair, identity of the mouse showed the behavior is discriminated with shaded or non-shaded background of the cell (e.g. W12 had 4 trials with submissive posture and all of them were acted by the same individual regardless of its winner or loser status). Four mice in three WT pairs, and three mice in three βERKO pairs showed the submissive posture.

Table S1

Target of the sniffing and grooming in the social interaction tests. The sniffing and grooming were categorized into face-targeted or body-targeted. Although sniffing and grooming were observed separately, they were analyzed collectively here because of relatively small number of grooming. Dominants often sniff and/or groom subordinates' face concurrently with barbering behavior (Long, 1972; Sarna et al., 2000) which is corresponding to dominant-subordinate relationship observed in social interaction (Wang et al., 2011). When both face-targeted and body-targeted sniffing/grooming occurred continuously during one behavioral event, it was counted as a face-targeted. Total number of the face-targeted and body-targeted sniffing and/or grooming in each genotype, rank determined by the tube test (Winner or Loser in the second column), and trial was cumulated and then probability of the face-targeted sniffing/grooming was compared. Statistical analysis was conducted using a Fischer's exact test, with stratified analysis of Benjamini and Hochberg method. In both WT and βERKO pairs, there was no significant winner-loser difference in the probability of face-targeted sniffing/grooming (WT and βERKO, trials 1, 2, 3, and 4, rank: n.s.). Moreover, there was no overall genotype difference in the probability of face-targeted sniffing/grooming when winner and loser were combined (trials 1, 2, 3, and 4, genotype: n.s.).

		WT		βERKO	
	Target	Body	Face	Body	Face
Trial 1	Winner	37	29	20	34
	Loser	37	29	22	20
Trial 2	Winner	22	12	28	29
	Loser	26	36	36	28
Trial 3	Winner	31	25	23	31
	Loser	27	30	31	21
Trial 4	Winner	24	25	23	13
	Loser	21	18	32	23

References

Long, S.Y. (1972). Hair-nibbling and whisker-trimming as indicators of social hierarchy in mice. *Anim. Behav.* 20**,** 10-12. doi:10.1016/S0003-3472(72)80167-2

Sarna, J.R., Dyck, R.H., and Whishaw, I.Q. (2000). The Dalila effect: C57BL6 mice barber whiskers by plucking. *Behav. Brain Res.* 108**,** 39-45. doi:10.1016/S0166-4328(99)00137-0
